# Elastic Moduli of Non-Chiral Singe-Walled Silicon Carbide Nanotubes: Numerical Simulation Study

**DOI:** 10.3390/ma15228153

**Published:** 2022-11-17

**Authors:** Nataliya A. Sakharova, André F. G. Pereira, Jorge M. Antunes

**Affiliations:** 1 Centre for Mechanical Engineering, Materials and Processes (CEMMPRE), Department of Mechanical Engineering, University of Coimbra, Rua Luís Reis Santos, Pinhal de Marrocos, 3030-788 Coimbra, Portugal; 2 Polytechnic Institute of Tomar, Quinta do Contador, Estrada da Serra, 2300-313 Tomar, Portugal

**Keywords:** silicon carbide nanotubes, Young’s and shear moduli, modelling, numerical simulation

## Abstract

Silicon carbide nanotubes (SiCNTs) have generated significant research interest due to their potential use in the fabrication of electronic and optoelectronic nanodevices and biosensors. The exceptional chemical, electrical and thermal properties of SiCNTs are beneficial for their application in high-temperature and harsh-environments. In view of the limited thermal stability of carbon nanotubes, they can be replaced by silicon carbide nanotubes in reinforced composites, developed for operations at high temperatures. However, fundamentally theoretical studies of the mechanical properties of the silicon carbide nanotubes are at an early stage and their results are still insufficient for designing and exploiting appropriate nanodevices based on SiCNTs and reinforced composites. In this context, the present study deals with the determination of Young’s and shear moduli of non-chiral single-walled silicon carbide nanotubes, using a three-dimensional finite element model.

## 1. Introduction

For nearly two decades, non-carbon nanotubes (N-CNTs) have been the focus of interest for the National Aeronautics and Space Administration (NASA) due to their use in hazardous environments [1]. Silicon carbide nanotubes (SiCNTs) have been sought after by NASA because of their excellent thermal resistance and durability under high temperatures in comparison to carbon nanotubes (CNTs). While the thermal stability of the CNTs is limited to 600 °C [2], the SiCNTs remain stable at higher temperatures, up to 800–1000 °C [1]. This high thermal stability makes SiCNTs viable for developing new devices for exploration in aggressive environments, such as in space missions. In addition, the SiCNTs have promising applications as biosensors [3] and toxic gas detectors [4].

SiCNTs were synthesized for the first time by Sun et al. [5], who converted multi-walled carbon nanotubes (MWCNTs) using a chemical reaction with silicon oxide. Later, SiCNTs were produced via thermally induced synthesis, with MWCNTs as a template [6] and through a controllable two-stage thermal process, using ZnS nanowires as models [7]. Pei et al. [8] grew SiCNTs through the hydrothermal method, firstly by synthesizing silicon nanotubes (SiNTs) and then by introducing the carbon atom, C, into the SiNTs by diffusion. More recently, Tony et al. [9] synthetized SiCNTs by microwave heating of silicon dioxide MWCNTs.

The reinforcement of composites for machinery parts by CNTs significantly improved their performance and helped to reduce the weight of its components. Nevertheless, this may not meet the requirements for numerous applications, such as high-temperature and high-power electronics, systems with improved thermal conductivity and nanodevices operating at high temperatures and in harsh environments. SiCNTs with optimum thermal properties, high conductivity and promising prospects for mass production can be suitable candidates to replace the CNTs. However, the silicon atom, Si, has a larger atomic radius, lower electronegativity and weaker bonds, which leads to properties of SiCNTs that are different from those of CNTs [10]. Regarding the SiCNTs mechanical properties, few studies have been conducted and existing studies have focused almost exclusively on theoretical works. As in the determination of the mechanical properties of CNTs, three classes of theoretical methods were used for this purpose in the case of SiCNTs, namely: the atomistic approach, involving ab initio and molecular dynamics (MD); the continuum mechanics (CM) approach; and the nanoscale continuum modelling (NCM), also called the molecular structural mechanics (MSM) approach. Although the atomistic approach provides good predictions of the mechanical properties, it requires a large computation cost and a demanding mathematical formulation [11].

On atomistic approaches, in the work by Baumeier et al. [12], the surface Young’s modulus (product of Young’s modulus by the nanotube wall thickness) of SiCNTs was assessed by ab initio density functional theory (DFT) calculations. With respect to MD, the key is to choose an appropriate potential, analytical or empirical, function to describe the interactions between atoms in the nanotubes (NTs). Moon et al. [13], Setoodeh et al. [14], Pan and Si [15] and Zhou et al. [16] used molecular dynamic simulations with Tersoff potential to describe the interactions between silicon (Si) and carbon (C) atoms, which allow the calculation of the Young’s modulus [13,14,15,16], studying the buckling behaviour under axial compression [14] and the tensile behaviour [15]. Le [17] used MD simulation with harmonic force fields to obtain an explicit expression for the SiCNTs surface Young’s modulus.

With regard to the CM approach, where the whole nanotube is replaced by a single continuum structure, Mercan and Civalek [18] analyzed the buckling behaviour of SiCNTs, using the continuum model based on the Euler-Bernoulli beam theory.

In contrast to CM, the NCM/MSM approach considers the bonds between Si and C atoms as elastic beams, making use of the connection between the nanotube molecular structure and solid mechanics. Genoese et al. [19] evaluated the surface Young’s and shear moduli of SiCNTs, using a link between the “stick-and-spring” (NCM/MSM) and the continuum thin shell Donnell (CM) models. Jiang and Guo [20] also used the “stick-and-spring” model and suggested an analytical solution for the surface Young’s modulus of SiCNTs. Ansari et al. [21] used the beam element to replace the Si-C bond, under the NCM/MSM approach, to study the buckling behaviour of SiCNTs.

The goal of this study is to assess the Young’s and shear moduli of non-chiral single-walled silicon carbide nanotubes (SWSiCNTs) with chiral indices and diameters in a broad range, making use of the NCM/MSM approach, which employs beam elements. So far, the NCM/MSM approach is the most commonly indicated for effective and fast computational simulation of the N-CNTs mechanical response. To this end, a three-dimensional numerical model was used, which allowed the determination of bending, tensile and torsional rigidities, and, afterwards, the calculation of the elastic moduli of SWSiCNTs.

## 2. Materials and Methods

### 2.1. Atomic Structure of SWSiCNTs

**Figure 1:**
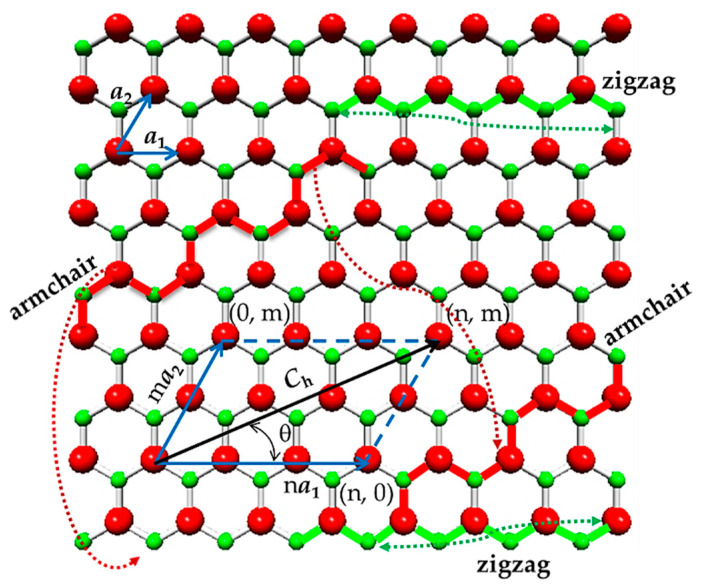
SiC hexagonal sheet with definitions of the chiral indices, n and m, chiral vector, **C_h_**, and chiral angle, θ; scheme for rolling up zigzag and armchair NTs configurations is illustrated. The Si atoms are shown in red; the C atoms in green.

As shown in Figure 1, the atomic structure of the hexagonal silicon carbide sheet is characterized by the chiral vector, **C_h_**, and the chiral angle, θ, given by the following expressions, respectively:

(1) $$C_{h}=na_{1}+ma_{2},$$

(2) $${\theta =\sin}^{-1}\frac{\sqrt[{}]{3}}{2}\frac{m}{\sqrt[{}]{n^{2}{+\mathrm{nm}+m}^{2}}},$$

where n and m are the chiral indices, both with integers values; $a_{1}$ and $a_{2}$ are the unit vectors of the hexagonal SiC lattice.

**Figure 2:**
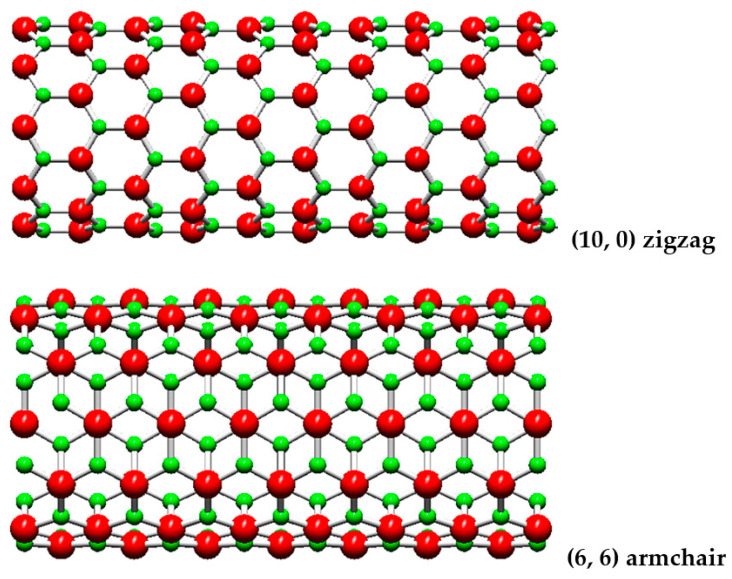
Configurations of (10, 0) zigzag and (6, 6) armchair SWSiCNTs, obtained using the software Nanotube Modeler©. The Si atoms are shown in red; the C atoms in green.

The SiC sheet can be rolled up into a cylinder in different ways, varying the chiral angle, θ, from 0° to 30° (see, Figure 1), forming single-walled nanotubes (NTs). When θ = 0° (m = 0) and θ = 30° (n = m), the resulting structures are called (n, 0) zigzag and (n, n) armchair NTs, respectively. These two limiting cases, which are schematically presented in Figure 2, constitute the group of non-chiral nanotubes. The configurations, which arise in the rest of the cases, when 0° < θ < 30° (n ≠ m), belong to the symmetry group called (n, m) chiral NTs.

NTs are characterized by the nanotube circumference, L_c_, and the diameter, $D_{n}$, expressed as follows:

(3) $$L_{c}=\left|C_{h}\right|=a\sqrt{n^{2}{+\mathrm{nm}+m}^{2}},$$

(4) $$D_{n}=\frac{L_{c}}{\pi }=\frac{a_{\mathrm{Si}\text{-}C}\sqrt{3\left(n^{2}{+\mathrm{nm}+m}^{2}\right)}}{\pi },$$

where a is the length of the unit vector, a, of the SiC lattice, defined through the length of the equilibrium Si-C covalent bond, $a_{\mathrm{Si}\text{-}C}$, as $a=\sqrt{3}a_{\mathrm{Si}\text{-}C}$. For the Si-C bond length, several values can be found in the literature, such as 0.177 nm [22], 0.179 nm [13] and 0.185 nm [23].

### 2.2. Geometric Characteristics of the SWSiCNTs and FE Modelling

The nanoscale continuum modelling/molecular structural mechanics approach was used, which substitutes the Si–C bonds of SWSiCNTs by equivalent beam elements. Li and Chou [24] established relationships between the tensile, E_b_A_b_, bending, E_b_I_b_, and torsional, G_b_J_b_, rigidities of beam elements, constituting the equivalent continuum structure, and the bond stretching, k_r_, bond bending, k_θ_, and torsional resistance, k_τ_, force constants, which describe the molecular structure:

(5) $$E_{b}A_{b}=lk_{r},\;E_{b}I_{b}=lk_{\theta },\;G_{b}J_{b}=lk_{\tau },$$

where *l* is the beam length.

Thus, Equation (5) is the basis for the analysis of the elastic behaviour of SWSiCNTs, using the link between the continuum and molecular mechanics, which together with the assumption of equivalence between the beam length, *l*, and the bond length, $a_{\mathrm{Si}\text{-}C}$, constitute the input data for the FE model (Table 1).

The FE models of the SiC nanotubes use the coordinates of the Si and C atoms to create the nodes and the appropriate connections between the nodes to generate the beam elements. The respective meshes were constructed using the Nanotube Modeler© software (version 1.8.0, ©JCrystalSoft), which produces program database files. These files contain the atom positions and interatomic connections, which serve as input to FE’s commercial code, ABAQUS^®^ (Abaqus 2020, Dassault Systèmes^®^). To transform the program database files, provided by the Nanotube Modeler© software, to a format suitable to be used in the commercial ABAQUS^®^ code, the home programme *InterfaceNanotubes.NM* was used [25]. Table 2 shows the geometric parameters of the non-chiral (zigzag and armchair) SWSiCNTs used in the present FE analyses. The length of nanotube was about 30 times greater than its diameter; this ensures that the elastic behaviour of NTs does not depend on the NTs length.

The mechanical behaviour of SWSiCNTs under numerical bending, tensile and torsion tests was studied with to the FE code ABAQUS^®^. Therefore, in the respective conventional tests, the transverse force, F_t_, the axial tensile force, F_a_, and the torsional moment, T, were applied to one edge of the NT, while the other edge is constrained. To carry out the torsion test, the loaded nodes were prevented from moving in the radial direction.

The axial displacement, u_a_, the transverse displacement, u_t_, and the twist angle, φ, are obtained from the FE analysis of the tensile, bending and torsion tests, respectively. Consequently, the tensile, EA, bending, EI, and torsional, GJ, rigidities of the SWSiCNTs can be determined as follows, respectively:

(6) $$\mathrm{EA}=\frac{F_{a}L_{n}}{u_{a}},$$

(7) $$\mathrm{EI}=\frac{F_{t}L_{n}^{3}}{{3u}_{t}},$$

(8) $$\mathrm{GJ}=\frac{{\mathrm{TL}}_{n}}{\varphi },$$

where L_n_ is the NT’s length.

### 2.3. Young’s and Shear Moduli of SWSiCNTs

As for the single-walled carbon [26,27], boron nitride [25] and phosphide [28] nanotubes, the Young’s, E, and shear, G, moduli of SWSiCNTs can be evaluated resourcing to the values of tensile, EA, bending, EI, and torsional, GJ, rigidities. The SWSiCNT can be considered as an equivalent hollow cylinder with the mean diameter $\bar{D}$ and the wall thickness, $t_{n}$. Their cross-sectional area, A, moment of inertia, I, and the polar moment of inertia, J, are given, respectively, by the following expressions:

(9) $$A=\frac{\pi }{4}\left[{\left(\bar{D}+t_{n}\right)}^{2}-{\left(\bar{D}-t_{n}\right)}^{2}\right]=\pi \bar{D}t_{n},$$

(10) $$I=\frac{\pi }{64}\left[{\left(\bar{D}+t_{n}\right)}^{4}-{\left(\bar{D}-t_{n}\right)}^{4}\right]=\frac{\pi {\bar{D}}^{3}t_{n}}{8}\left[1+{\left(\frac{t_{n}}{\bar{D}}\right)}^{2}\right],$$

(11) $$J=\frac{\pi }{32}\left[{\left(\bar{D}+t_{n}\right)}^{4}-{\left(\bar{D}-t_{n}\right)}^{4}\right]=\frac{\pi {\bar{D}}^{3}t_{n}}{4}\left[1+{\left(\frac{t_{n}}{\bar{D}}\right)}^{2}\right]$$

Knowing the EA and EI rigidities and using Equations (9) and (10), the diameter $\bar{D}$ can be calculated as follows:

(12) $$\frac{\mathrm{EI}}{\mathrm{EA}}=\frac{1}{8}\left({\bar{D}}^{2}+t_{n}^{2}\right)⟹\bar{D}\sqrt{8\left(\frac{\mathrm{EI}}{\mathrm{EA}}\right)-t_{n}^{2}}.$$

Subsequently, replacing the mean diameter, $\bar{D}$, by expression (12) in Equations (9) and (11), it is possible to calculate the E and G moduli using the following expressions, respectively:

(13) $$E=\frac{\mathrm{EA}}{A}=\frac{\mathrm{EA}}{\pi t_{n}\sqrt{8\left(\frac{\mathrm{EI}}{\mathrm{EA}}\right)-t_{n}^{2}}},$$

(14) $$G=\frac{\mathrm{GJ}}{J}=\frac{\mathrm{GJ}}{2\pi t_{n}\left(\frac{\mathrm{EI}}{\mathrm{EA}}\right)\sqrt{8\left(\frac{\mathrm{EI}}{\mathrm{EA}}\right)-t_{n}^{2}}}.$$

As in the case of most N-CNTs [29], there is uncertainty with regard to the NT wall thickness of SiCNTs. The multi-walled SiCNTs synthesized by Sun et al. [5] had the interlayer spacing in the range of 0.35 to 0.45 nm, which is different from 0.34 nm, whose value corresponds to the graphite interlayer spacing and is commonly used as the wall thickness, $t_{n}$, of the carbon and boron nitride NTs. Thus, in the present study, for the purpose of comparison, the E and G moduli of the SWSiCNTs were calculated for $t_{n}$ = 0.34, 0.39, 0.45 nm.

As the determination of the SWSiCNTs Young’s, E, and shear, G, moduli, according to Equations (13) and (14), requires reliable knowledge of the value of t_n_, several authors have chosen to report the values of the surface Young’s ($E_{s}{=\mathrm{Et}}_{n}$) and shear ($G_{s}{=\mathrm{Gt}}_{n}$) moduli, $E_{s}$ and $G_{s}$ of the SWSiCNTs, which can be assessed, respectively, as follows:

(15) $$E_{s}={\mathrm{Et}}_{n}=\frac{\mathrm{EA}}{\pi \sqrt{8\left(\frac{\mathrm{EI}}{\mathrm{EA}}\right)-t_{n}^{2}}},$$

(16) $$G_{s}=Gt_{n}=\frac{\mathrm{GJ}}{2\pi \left(\frac{\mathrm{EI}}{\mathrm{EA}}\right)\sqrt{8\left(\frac{\mathrm{EI}}{\mathrm{EA}}\right)-t_{n}^{2}}}.$$

The viability of Equations (15) and (16), to be used to evaluate the $E_{s}$ and $G_{s}$ moduli, is based on the fact that the value of $t_{n}^{2}$ is small and does not significantly influence the results. Nevertheless, to verify that this assumption is correct, the reduced surface Young’s, $E_{s}^{*}$, and shear, $G_{s}^{*}$, moduli were calculated, using the following expressions, respectively:

(17) $$E_{S}^{*}=\frac{\mathrm{EA}}{\pi \sqrt{8\left(\frac{\mathrm{EI}}{\mathrm{EA}}\right)}},$$

(18) $$G_{S}^{*}=\frac{\mathrm{GJ}}{2\pi \left(\frac{\mathrm{EI}}{\mathrm{EA}}\right)\sqrt{8\left(\frac{\mathrm{EI}}{\mathrm{EA}}\right)}}.$$

## 3. Results and Discussion

### 3.1. Rigidities of SWSiCNTs

**Figure 3:**
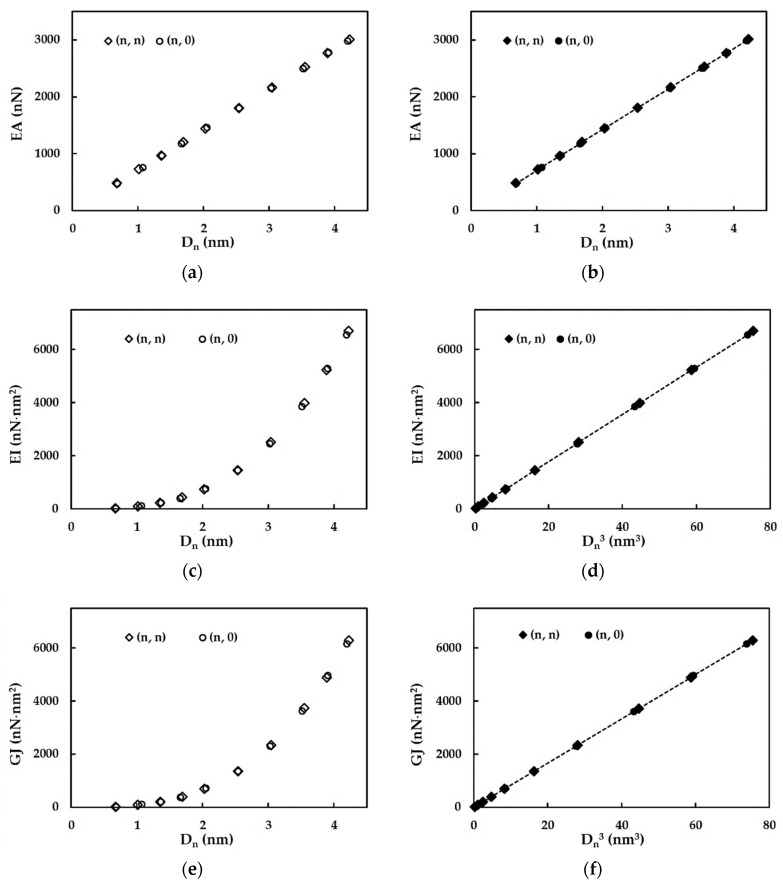
Evolutions of (**a**,**b**) tensile, EA, (**c**) bending, EI, and (**e**) torsional, GJ, rigidities as a function of the nanotube diameter, $D_{n}$; (**d**) bending, EI, and (**f**) torsional, GJ, rigidities and as a function of $D_{n}^{3}$ for non-chiral SWSiCNTs. The results refer to the nanotubes in Table 2.

The tensile, EA, bending, EI, and torsional, GJ, rigidities of non-chiral SWSiCNTs, from Table 2, calculated with Equations (6)–(8), are presented in Figure 3a,c,e, respectively, as a function of the NT diameter, $D_{n}$. As it was already established by the authors for the cases of the chiral and non-chiral single-walled carbon nanotubes (SWCNTs) [26,27], the single-walled boron nitride nanotubes (SWBNNTs) [25], and the phosphide NTs [28], for non-chiral SWSiCNTs, the EA values can be represented by a linear function of the NT diameter, $D_{n}$ (see, Figure 3b), and the EI and GJ values can be described with the help of a linear function of $D_{n}^{3}$ (see, Figure 3d,f, respectively).

As previously established by the authors for the SWCNTs [26,27], SWBNNTs [25] and phosphide NTs [28], as well as in the current case of SWSiCNTs, the behaviour is characterized by the straight lines in Figure 3b,d,f and can be described by the following expressions:

(19) $${\mathrm{EA}=\alpha }_{\mathrm{SiC}}D_{n},$$

(20) $${\mathrm{EI}=\beta }_{\mathrm{SiC}}D_{n}{}^{3},$$

(21) $${\mathrm{GJ}=\gamma }_{\mathrm{SiC}}D_{n}{}^{3},$$

where $\alpha_{\mathrm{SiC}}$ = 711.59 nN/nm, $\beta_{\mathrm{SiC}}$ = 88.84 nN/nm and $\gamma_{\mathrm{SiC}}$ = 83.36 nN/nm are the fitting parameters.

It is worth noting that the linear function presented by Equation (19) and the cubic functions expressed by Equations (20) and (21) can be comprehended based on the quasi linear relationship of the cross-sectional area, A (Equation (9)), and the cubic relationships between the moment of inertia, I (Equation (10)), and the polar moment of inertia, J (Equation (11)) with the nanotube diameter, respectively.

**Figure 4:**
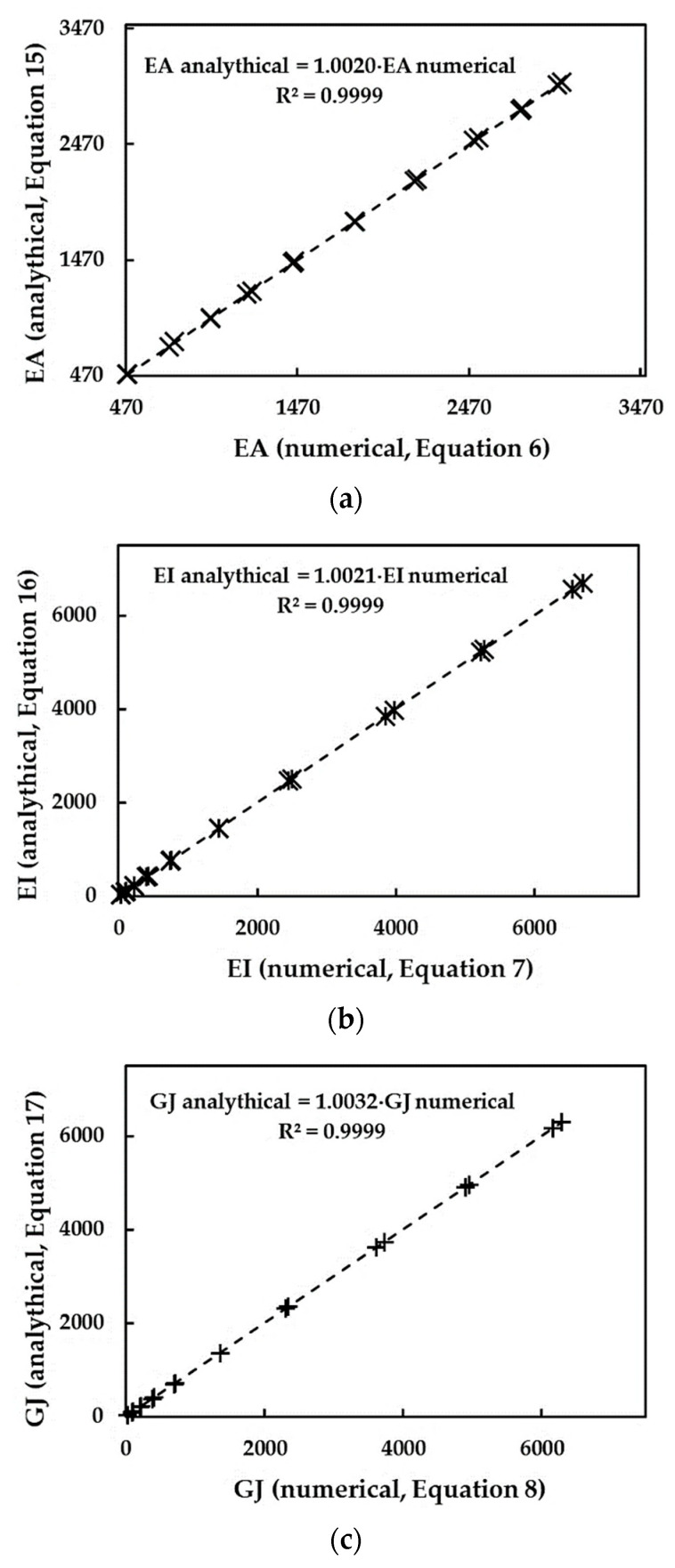
Comparison between the (**a**) tensile, EA, (**b**) bending, EI, and (**c**) torsional, GJ, rigidities, obtained from FE analyses with help of Equations (6)–(8), and those evaluated by analytical expressions (15)–(17).

To investigate the accuracy of the aforementioned analytical expressions for the evaluation of the three rigidities, Figure 4 compares the EA, EI and GJ rigidities obtained from FE analysis, using Equations (6)–(8), and those evaluated by Equations (19)–(21). As can be seen, the average difference between the EA, EI and GJ values acquired from FE analyses and those calculated analytically are 0.20%, 0.21% and 0.32% for EA, EI and GJ rigidities, respectively.

Substituting, in Equations (15)–(18), the tensile, EA, bending, EI, and torsional, GJ, rigidities by the respective expressions (19)–(21), and knowing the parameters $\alpha_{\mathrm{SiC}}$, $\beta_{\mathrm{SiC}}$ and $\gamma_{\mathrm{SiC}}$, the SWSiCNTs diameter, $D_{n}$, and the wall thickness, $t_{n}$, it is possible to calculate the Young’s and shear moduli:

(22) $$E=\frac{\alpha_{\mathrm{SiC}}D_{n}}{{\pi t}_{n}\sqrt{8\left(\frac{\beta_{\mathrm{SiC}}}{\alpha_{\mathrm{SiC}}}\right)D_{n}^{2}{-t}_{n}^{2}}},$$

(23) $$G=\frac{\gamma_{\mathrm{SiC}}D_{n}}{2\pi \left(\frac{\beta_{\mathrm{SiC}}}{\alpha_{\mathrm{SiC}}}\right)t_{n}\sqrt{8\left(\frac{\beta_{\mathrm{SiC}}}{\alpha_{\mathrm{SiC}}}\right)D_{n}^{2}{-t}_{n}^{2}}},$$

and the reduced surface Young’s and shear moduli, which are independent from $D_{n}$:

(24) $$E_{S}^{*}=\frac{\alpha_{\mathrm{SiC}}}{\pi \sqrt{8\left(\frac{\beta_{\mathrm{SiC}}}{\alpha_{\mathrm{SiC}}}\right)}},$$

(25) $$G_{S}^{*}=\frac{\gamma_{\mathrm{SiC}}}{\pi \sqrt{32{\left(\frac{\beta_{\mathrm{SiC}}}{\alpha_{\mathrm{SiC}}}\right)}^{3}}}.$$

As a result, Equations (22)–(25) allow the assessment of the SWSiCNTs elastic moduli without resorting to numerical simulation.

### 3.2. Young’s Modulus of SWSiCNTs

**Figure 5:**
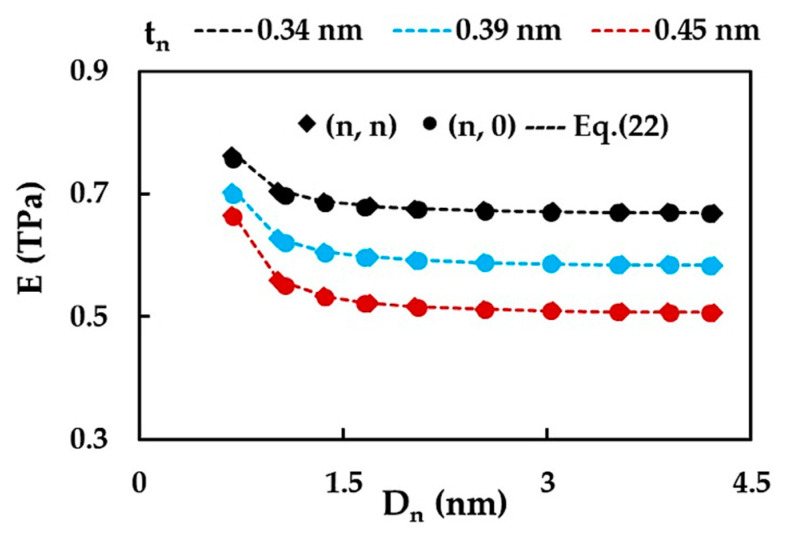
Evolutions of the Young’s modulus, E, with the NT diameter, $D_{n}$, for SWSiCNTs, considering the NT wall thicknesses, $t_{n}$ = 0.34 nm, 0.39 nm and 0.45 nm.

First, the results of the non-chiral SWSiCNTs Young’s modulus, calculated with Equations (13) and (22), for three different values of the NT wall thickness, $t_{n}$, are examined. In Figure 5, the evolutions of the Young’s modulus, E, with the NT diameter, $D_{n}$, for the cases of $t_{n}$ = 0.34, 0.39, 0.45 nm, are shown. Whatever the $t_{n}$ value and the NT symmetry group, whether zigzag or armchair, the Young’s modulus initially decreases with $D_{n}$ and then tends to stabilize for the NT diameters $D_{n}$ > 1.65 nm. This decrease is more pronounced when the NT wall thickness is greater (see, Figure 5). It should be noted that Equation (22) allows the evaluation of the Young’s modulus of SWSiCNTs with satisfactory accuracy, regardless of the chiral indices and diameter, and without the need to resort to numerical simulation. The values for which the E of the SWSiCNTs converges, progressively decrease with the increase in $t_{n}$, and are E = 0.670, 0.585 and 0.508 TPa, for $t_{n}$ = 0.34, 0.39, 0.45 nm, respectively.

**Figure 6:**
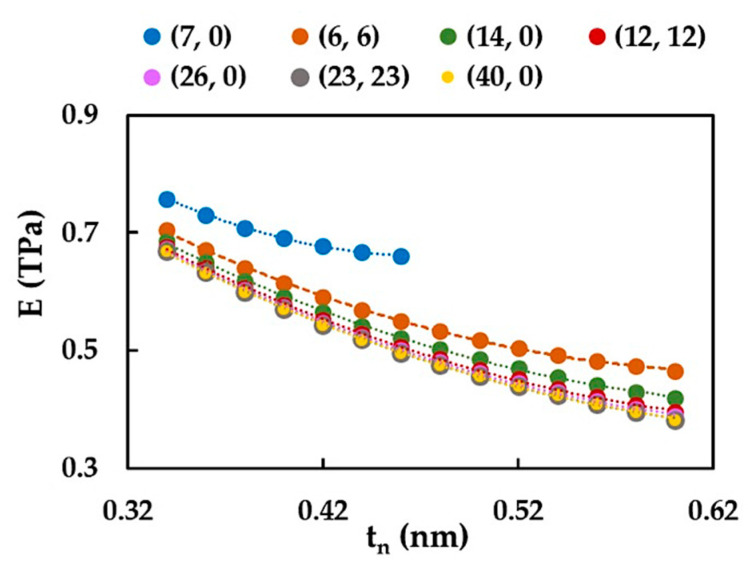
Evolutions of the Young’s modulus, E, with the NT wall thickness, $t_{n}$ for selected SWSiCNTs from Table 2.

The results related to the effect of the nanotube wall thickness, $t_{n}$, on the Young’s modulus, E, presented in Figure 5, put forward analyses of the evolutions of E as a function of $t_{n}$ in the range of 0.34 nm to 0.60 nm, as it is shown in Figure 6 for selected SWSiCNTs from Table 2.

It can be concluded that the Young’s modulus, E, decreases when the wall thickness, $t_{n}$, increases, and the decreasing rate of E slows down when the SWSiCNTs diameter, $D_{n}$, decreases. These results can be useful to facilitate the comparison with the values of E available in the literature and to make assumptions about a viable value of the SWSiCNT wall thickness. This approach was used to compare the current Young’s modulus results with those assessed by other authors.

**Figure 7:**
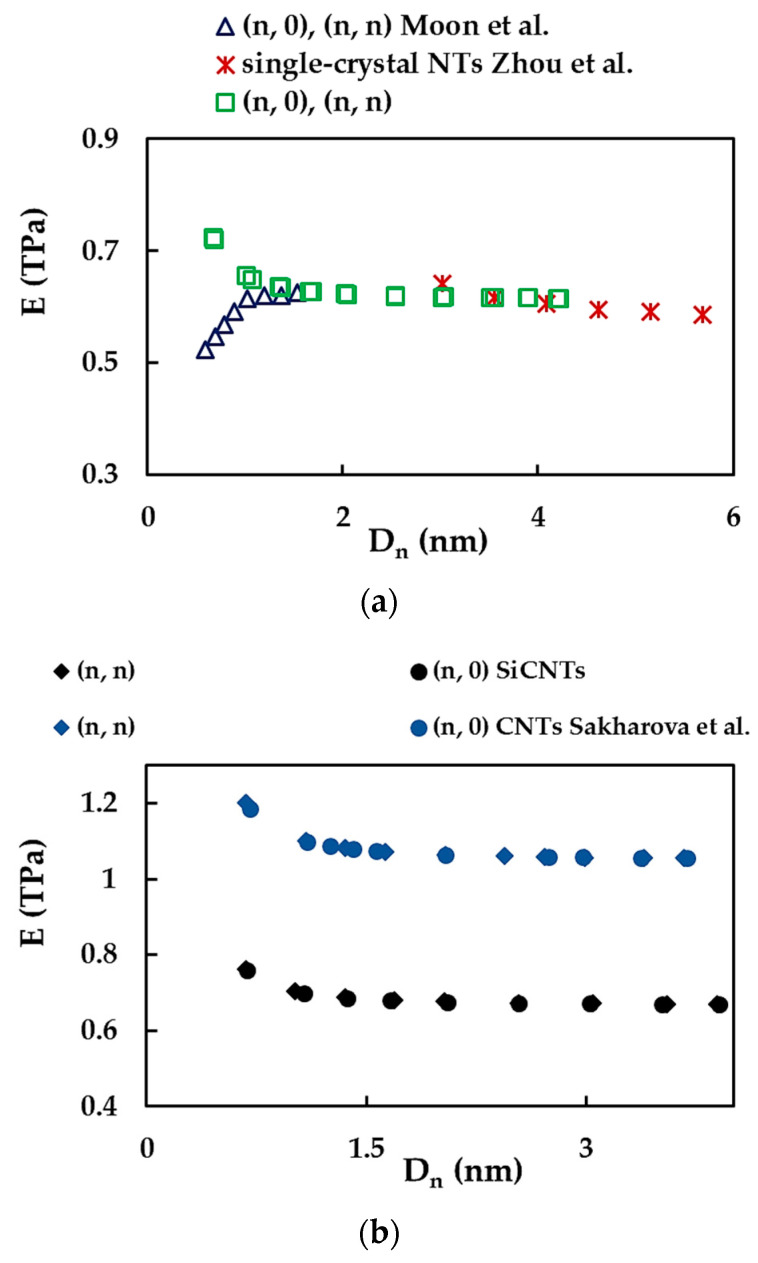
(**a**) Comparison of the current Young’s modulus, E, results calculated for the wall-thickness $t_{n}$ = 0.37 nm with those by Moon et al. [13] and Zhou et al. [16]; (**b**) comparison of the E values of SWSiCNTs with those of SWCNTs [25], for $t_{n}$ = 0.34 nm in both cases.

Figure 7 compares the current Young’s modulus results with those available in the literature for SiCNTs (Figure 7a) and with those of SWCNTs obtained by the authors in previous studies (Figure 7b). To our knowledge, the Young’s modulus values of SiCNTs were reported in two studies [13,16], both employing MD simulations with Tersoff potential. Moon et al. [13], who obtained a single trend for (n, n) and (n, 0) SWSiCNTs, found that the Young’s modulus increases for small NTs diameter, $D_{n}$, and then tends to reach an approximately constant value. On the other hand, according to Zhou et al. [16], the value of E for monocrystalline SiCNTs decreases insignificantly at the beginning of the evolution trend and then becomes stable when the $D_{n}$ increases. Using data from Figure 6, better agreement was obtained with the results from the literature for the Young’s modulus, E, when the value of the NT wall thickness, $t_{n}$, is equal to 0.37 nm (see, Figure 7a). Thus, Equation (22) gives E at approximately 0.620 TPa for NTs with diameters in the range of 1.350 nm to 4.220 nm, which is comparable to the value of E obtained by of Moon et al. [13] and Zhou et al. [16]. The comparison shown in Figure 7b illustrates that the Young’s modulus of the SWSiCNTs is approximately 37% lower than that calculated for the SWCNTs. This should be taken into account when SiCNTs are considered to replace CNTs in applications and devices, especially those where high mechanical resistance of the components is required, such as NTs-reinforced ceramics operating in aggressive environments.

**Figure 8:**
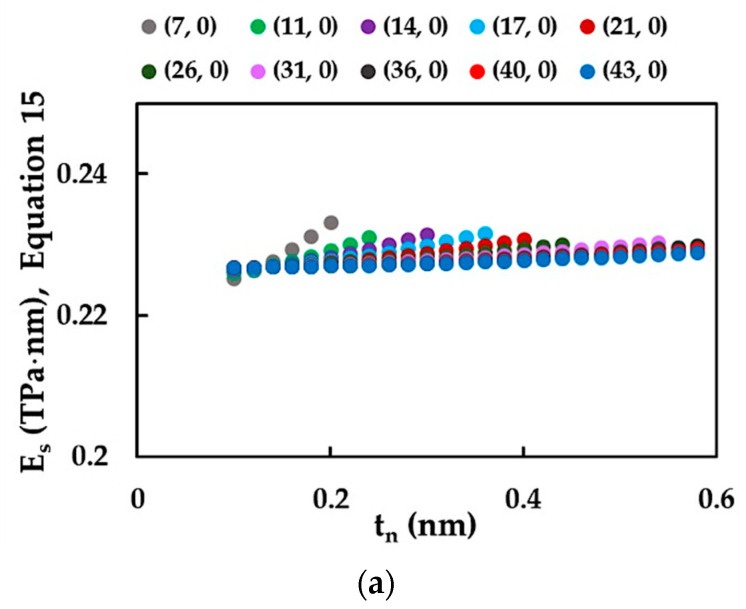

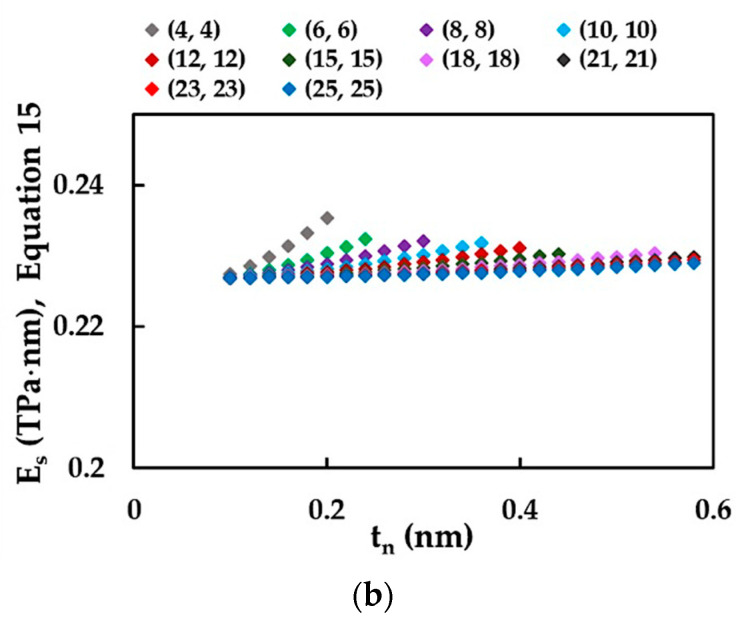
Evolution of the surface Young’s modulus, $E_{S}$, calculated with help of Equation (15), as a function of the wall thickness,${\;t}_{n}$, for (**a**) zigzag and (**b**) armchair SWSiCNTs.

As there is no reported accurate value of $t_{n}$ for SWSiCNTs, surface Young’s modulus, $E_{S}$, results are predominantly available in the literature; these were also calculated in the present study. First, the $E_{S}$ values for zigzag and armchair nanotubes were evaluated by Equation (15) as a function of the NT wall thickness and analyzed for $t_{n}$ in the range of 0.1 nm to 0.6 nm (Figure 8a,b). For SWSiCNTs with diameters $D_{n}$ ≲ 1.00 nm, the surface Young’s modulus, $E_{S}$, increases with the increasing wall thickness, $t_{n}$, with the $E_{S}$ value nearly constant at the beginning of the trend. For the SWSiCNTs with larger diameters, $E_{S}$ remains approximately constant (i.e., is nearly independent of the wall thickness) for higher $t_{n}$ values. The larger the NT diameter, $D_{n}$, the larger the value of $t_{n}$ for which the surface Young’s modulus starts to increase and becomes dependent on the wall thickness (see, Figure 8a,b). From Figure 8a,b it can be concluded that the mechanical behaviour of SWSiCNTs with a wall thickness in the range $t_{n}\;≳D_{n}/5$ can be understood as that of solid cylinders and not as hollow thin-walled tubes. This explains the Young’s modulus results showed in Figure 5, i.e., the increase in the Young’s modulus, E, of the SWSiCNTs with small diameters $D_{n}$ < 1.35 becomes more pronounced for larger values of $t_{n}$.

The results presented in Figure 8 were used as the base of the method to assess the surface Young’s modulus, $E_{S}$, of the non-chiral SWSiCNTs. For the analyses, only the horizontal portions of the evolutions $E_{S}=f\;\left(t_{n}\right)$, plotted in Figure 8, were taken into consideration. Throughout these horizontal portions, which approximately correspond to the nanotube wall thickness range $t_{n}\;≲{\;D}_{n}/5$, $E_{S}$ remains nearly constant and independent from $t_{n}$. In this way, the average $E_{S}$ value, calculated based on those of the horizontal portion of the evolution $E_{S}=f\;\left(t_{n}\right)$, defines the surface Young’s modulus for each SWSiCNT under study. Looking at Figure 8a,b, it should be noted that the methodology described is less accurate when it is required to evaluate the surface Young’s modulus of NTs with small diameters.

**Figure 9:**
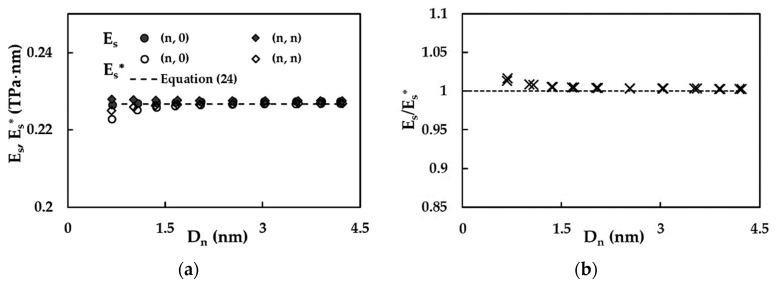
Evolutions of (**a**) the surface Young’s modulus, $E_{S}$, and the reduced surface Young’s modulus, $E_{S}^{*}$, and (**b**) the ratio $E_{S}$/$E_{S}^{*}$ as a function of the NT diameter, $D_{n}$, for zigzag and armchair SWSiCNTs (Table 2).

Figure 9a presents the evolution of the surface Young’s modulus, $E_{S}$, obtained by the methodology described above, and the reduced surface of the Young’s modulus, $E_{S}^{*}$, determined using the Equation (17), with the NT diameter, $D_{n}$, for zigzag and armchair SWSiCNTs. The reduced surface of the Young’s modulus, $E_{S}^{*}$, calculated by Equation (24), is also shown for the purpose of comparison.

The surface Young’s modulus, $E_{S}$, assessed by the methodology proposed, is approximately constant over the entire range of diameters of the non-chiral SWSiCNTs studied. The reduced surface of the Young’s modulus, $E_{S}^{*}$, computed by Equation (17), slightly increases for small NTs diameters, $D_{n}$, and then the $E_{S}^{*}$ value is practically stable with the increase in $D_{n}$. At the beginning of the trend, the values of $E_{S}$ are higher than those calculated for $E_{S}^{*}$, then with the increase in the NT diameter, both the surface and the reduced surface of the Young’s moduli tend to possess nearly the same value, equal to that assessed by Equation (24) (see, Figure 9a). The latter is independent of the nanotube diameter and is defined only by the fitting parameters of Equations (19)–(21). It can be concluded that Equation (24) allows for the obtaining of accurate values of the surface of the Young’s modulus of the non-chiral SWSiCNTs with $D_{n}$ > 1.65 nm, without resorting to numerical simulation. The dissimilarity of the trends in the evolutions of the surface and the reduced surface of the Young’s moduli found for small NT diameters can be explained by the insufficient accuracy of the methodology proposed to evaluate $E_{S}$ for SWSiCNTs with $D_{n}\;≲$ 1.00 nm. As Figure 9b shows, the mean difference between the SWSiCNTs surface of the Young’s modulus, $E_{S}$, and their reduced surface of the Young’s modulus, $E_{S}^{*}$, is approximately 1.15% for NTs with diameters in range 0.676 nm $≲{\;D}_{n}\;≲$ 1.073 nm. After that, as the $D_{n}$ increases, the mean difference between $E_{S}$ and $E_{S}^{*}$ decreases and attains ≈ 0.31% for SWSiCNTs with $D_{n}$ ≳ 2.50 nm. Taking into account the results in Figure 9b, it can be noted that Equation (17), for the reduced Young’s modulus, $E_{S}^{*}$, permits the calculation of the SWSiCNTs surface of the Young’s modulus, $E_{S}$, with satisfactory accuracy over the entire $D_{n}$ range. The lower precision in the determination of $E_{S}$ values for nanotubes with $D_{n}\;≲$ 1.073 nm originates from the limitation of the proposed model for SWSiCNTs with small diameters. Thus, the surface of the Young’s modulus computed by Equation (17) was used hereinafter for comparison with literature results.

**Figure 10:**
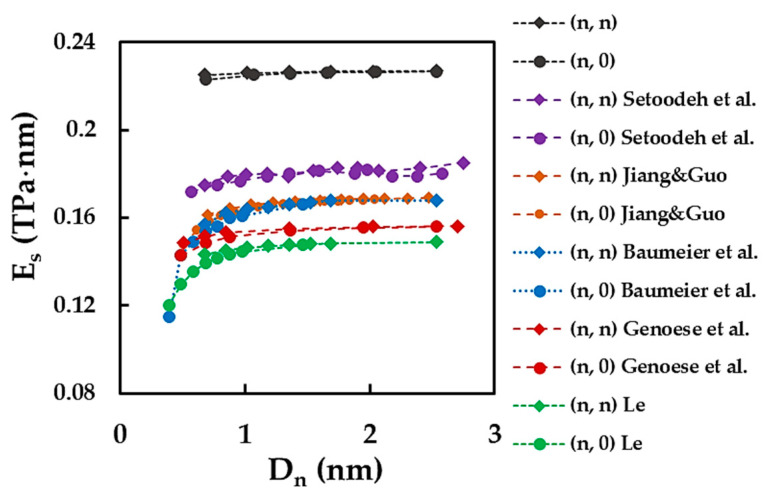
Comparison of the current results of the surface Young’s modulus of non-chiral SWSiCNTs with those reported in the literature [12,14,17,19,20], as a function of the NT diameter, $D_{n}$.

Figure 10 compares the current surface of the Young’s modulus results with those available in the literature. A considerable scattering of the $E_{S}$ values can be noticed in Figure 10. As already reported for CNNs [11] and N-CNTs [29], significant discrepancies in the elastic constants results occur due to different modelling approaches, potential functions used, and calculation methods employed. The surface of the Young’s modulus values reported so far are in the range 0.14 TPa·nm [17] $≲E_{S}≲$ 0.18 TPa·nm [14] which are between 52% and 22%, respectively, lower than the $E_{S}$ value calculated by Equation (17) in the present study. Regard the literature results from Figure 10, there is a very good concordance between $E_{S}$ assessed by Baumeier et al. [12], using ab initio DFT calculations, and Jiang and Guo [20], who employed an analytical solution, based on the “stick-and-spring” model under NCM/MSM approach, for this end. On the other hand, the $E_{S}$ values of Baumeier et al. [12] and Jiang and Guo [20] are at approximately 7% lower than the surface of the Young’s modulus of Setoodeh et al. [14], calculated using MD simulation. In turn, the value of $E_{S}$ evaluated in the MD simulation work of Le [17] is approximately 11% lower than that of Baumeier et al. [12] and Jiang and Guo [20]. Despite the studies of Genoese et al. [19] and Jiang and Guo [20] sharing the modelling approach, Genoese et al. [19] reported a surface of the Young’s modulus approximately 7% lower than that of Jiang and Guo [20], most likely due to the different force field constants and calculation methods used.

The discrepancy of the $E_{S}$ results presented in Figure 10 is due to the different modelling and calculation approaches used to evaluate the SWSiCNTs surface of the Young’s modulus. In order to facilitate comparative analyses, the results from Figure 10 are resumed in Table 3.

### 3.3. Shear Modulus of SWSiCNTs

In this section, the results of the shear modulus, G, and the surface shear modulus, $G_{S}$, of the non-chiral SWSiCNTs are analyzed within the same type of framework established for the Young’s and surface Young’s moduli in Section 3.2. To the best of our knowledge, studies to evaluate the SiCNTs shear modulus are uncommon and so far, $G_{S}$ values were reported only by Genoese et al. [19].

**Figure 11:**
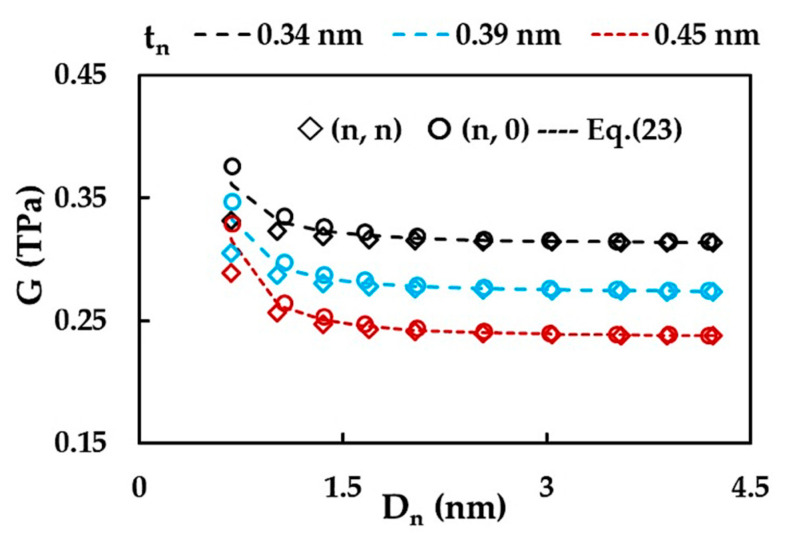
Evolutions of the shear modulus, G, with the NT diameter, $D_{n}$, for SWSiCNTs, considering the NT wall thickness, for t_n_ = 0.34 nm, 0.39 nm and 0.45 nm.

Figure 11 presents the evolution of the shear modulus, G, computed with help of Equations (14) and (23) as a function the NT diameter, $D_{n}$, for three different values of wall thickness, t_n_ = 0.34, 0.39, 0.45 nm.

The shear modulus, G, of the non-chiral SWSiCNTs increases for NT diameters, $D_{n}$ ≲ 1.1 nm, and for high $D_{n}$, G tends to attain a stable value, equal to that calculated by Equation (23). The converged average value of the shear modulus decreases when the wall thickness increases: the G value is 0.315, 0.275 and 0.239 TPa for $t_{n}$ = 0.34, 0.39, 0.45 nm, respectively. The G values for (n, n) armchair and (n, 0) zigzag are almost equal for $D_{n}$ > 1.6 nm, but for NT diameters in the range 0.67 ≲ $D_{n}$ ≲ 1.36 nm, the trends for the shear modulus evolution are clearly influenced by the chiral angle, θ, and differ between (n, 0) and (n, n) nanotubes. As can be seen in Figure 11, Equation (23) does not allow the calculation of accurate values of G of the (n, n) armchair SWSiCNTs with the diameters $D_{n}$< 1.6 nm.

Similar to the case of the surface of the Young’s modulus, the non-chiral SWSiCNTs surface of the shear modulus, $G_{S}$, calculated by Equation (16) was plotted as a function of the NT wall thickness, $t_{n}$, in the range of 0.1 nm to 0.6 nm, as shown in Figure 11a,b.

**Figure 12:**
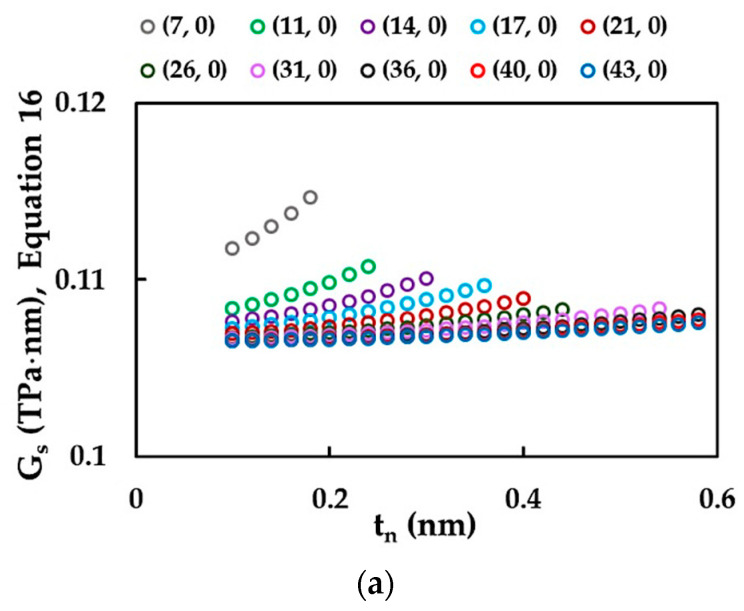

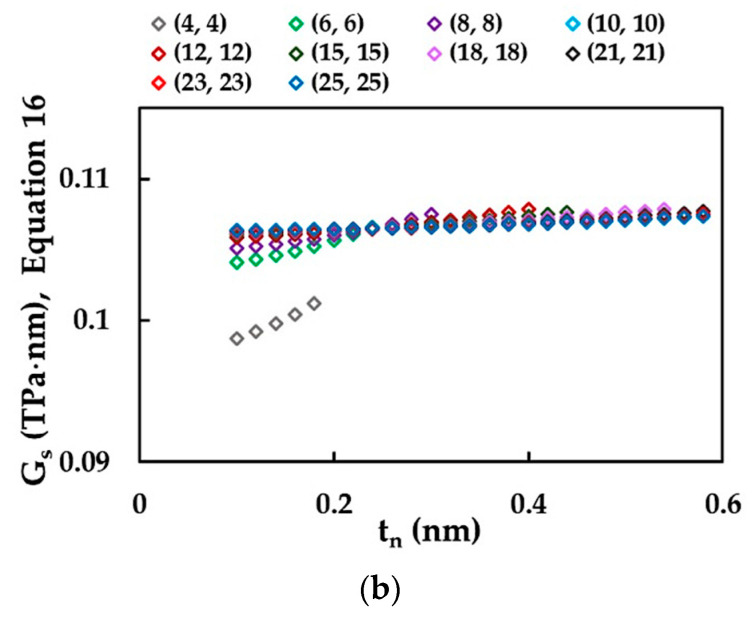
Evolutions of the surface shear modulus, $G_{S}$, calculated with help of Equation (16), as a function of the wall thickness,${\;t}_{n}$, for (**a**) zigzag and (**b**) armchair SWSiCNTs.

The $G_{S}$ values calculated by Equation (16) are almost independent from the wall-thickness $t_{n}$ ≲ $D_{n}$/5, and with increasing of $t_{n}$, the surface shear modulus increases. This trend in the evolution of $G_{S}$ as a function of the wall thickness is much less pronounced for the SWSiCNTs with small diameters $D_{n}$ ≲ 1.00 nm (see, Figure 12a,b). Thus, it can be concluded that the mechanical behaviour of the NTs deviates from that of the hollow tube when the value of $t_{n}$ is equal to one-fifth of the NT diameter. Similar to the case of the surface of the Young’s modulus, the methodology to assess the surface shear modulus consists of the calculation of the average $G_{S}$ value from those corresponding to the horizontal portions of the evolutions $G_{S}=f\;\left(t_{n}\right)$ as shown in Figure 11a,b.

**Figure 13:**
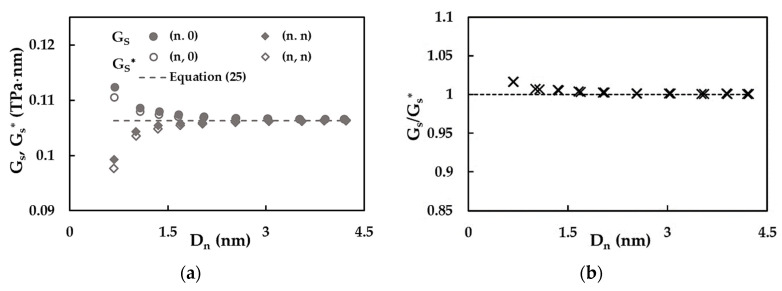
Evolutions of (**a**) the surface shear modulus, $G_{S}$, and the reduced surface shear´s modulus, $G_{S}^{*}$, and (**b**) the ratio $G_{S}$/$G_{S}^{*}$ as a function of the NT diameter, $D_{n}$, for zigzag and armchair SWSiCNTs (Table 2).

Figure 13a shows the evolutions of the surface of the shear modulus, $G_{S}$, evaluated with help of the methodology proposed and the reduced shear modulus, $G_{S}^{*}$, calculated by Equation (18), with the nanotube diameter, $D_{n}$. The evolutions of both surface and reduced surface of the shear moduli follow different trends when comparing the (n, 0) zigzag with the (n, n) armchair NTs.

The $G_{S}$ and $G_{S}^{*}$ values of the (n, 0) SWSiCNTs decrease for NT diameters $D_{n}$ ≲ 1.65 nm and both moduli stabilize with the increasing $D_{n}$ and converge to the value of $G_{S}^{*},$ calculated by Equation (25), which is independent to the NT diameter. For (n, n) SWSiCNTs, $G_{S}$ and $G_{S}^{*}$ increase for $D_{n}$ ≲ 1.65 nm and with of the increase in the NT diameter, the $G_{S}$ and $G_{S}^{*}$ values become nearly constant and, as in the case of (n, 0) NTs, converge to the reduced surface of the shear modulus, $G_{S}^{*}$, assessed by Equation (25). Thus, it can be concluded that Equation (25) permits the calculation of the surface of the shear modulus of SWSiCNTs with diameters $D_{n}$ > 1.65 nm, without resourcing to numerical simulation. It is worth noting that the values of $G_{S}$ and $G_{S}^{*}$ for (n, 0) NTs are greater than those for (n, n) NTs with diameters $D_{n}$ ≲ 2.00 nm; the greatest difference occurs for small diameters ($D_{n}$ ≲ 1.00 nm). These results are in agreement with the $G_{S}$ evolutions as a function of the NT wall thickness, shown in Figure 12a,b.

As can be seen from Figure 13b, the largest mean difference between $G_{S}$ evaluated by the methodology proposed and $G_{S}^{*}$ calculated by Equation (18), of 1.64%, occurs for the SWSiNTs with small diameters $D_{n}$ < 1.00 nm. The value of the ratio $G_{S}$/$G_{S}^{*}$ decreases with increasing $D_{n}$, and becomes equal to 0.13% for diameters $D_{n}$ ≳ 2.028 nm. As a result, Equation (18), for the reduced surface of the shear modulus, $G_{S}^{*}$, can be reliably used to calculate the surface of the shear modulus, $G_{S}$, giving accurate $G_{S}$ values, with the exception of the NTs with the diameters under 1.00 nm, for which the precision of Equation (18) is smaller but still acceptable.

**Figure 14:**
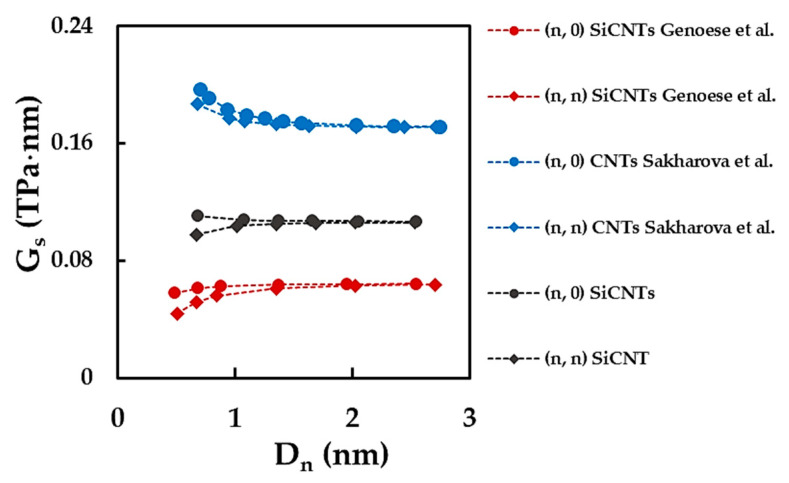
Comparison of the current results of the surface shear modulus, $G_{S}$, of non-chiral SWSiCNTs with those reported in the literature for SWSiCNTs [19] and SWCNTs [25]. In the case of the SWCNTs, $G_{S}$ was calculated using the shear modulus, G, results and assuming $t_{n}$ = 0.34 nm.

Figure 14 compares the current shear modulus results, obtained by Equation (18), and those available in the literature for SWSiCNTs [19] and SWCNTs [25].

The average value to which the surface of the shear modulus of the non-chiral SWSiCNTs converges is approximately 36% lower than that evaluated for the SWCNTs. This should be taken into account in the design and construction of NTs-based devices and systems, where SiCNTs are considered a replacement for CNTs. Regarding the $G_{S}$ results available in literature for the SWSiCNTs, the only possible comparison can be made with those of Genoese et al. [19]. Reasonable agreement is observed when the trends of the evolutions of the surface of the shear modulus for (n, 0) and (n, n) nanotubes are considered. Similar to the present study, Genoese et al. [19] reported the values of $G_{S}$ of (n, 0) zigzag NTs higher than those of (n, n) armchair NTs for diameters $D_{n}$ < 1.00 nm; with the increasing $D_{n}$, the surface shear modulus converges to a unique value in both cases (see, Figure 14). However, this converged average value is approximately 40% lower than the $G_{S}$ currently calculated. Despite the study of Genoese et al. [19], which used the NCM/MSM modelling approach with a “stick-and-spring” model and nearly the same force field constants, the methods for assessing $G_{S}$ differ, as Genoese et al. [19] assumed a continuum thin shell model to calculate the surface of the shear modulus.

## 4. Conclusions

The Young’s and shear moduli of non-chiral SWSiCNTs were assessed using numerical simulation, based on the NCM/MSM approach. The main achievements of the present study are presented in the following paragraphs.

Equations establishing the relationship between each of the three rigidities—tensile, bending and torsional—and the NT diameter were obtained. The fitting parameters of Equations (19)–(21), which permit the assessment of the rigidities of the SiC nanotubes—regardless of the symmetry group: zigzag or armchair—were calculated. In this way, the previously established method for calculating the three rigidities without resourcing to numerical simulation, is extended to silicon carbide NTs.

The evolutions of the Young’s modulus with the nanotube wall thickness were used to make assumptions regarding the realistic value of $t_{n}$, and to enable comparison with the results available in the literature.

The accuracy of Equations (17) and (18), for the evaluation of the surface of the Young’s and shear moduli, respectively, was demonstrated. In our view, these equations are suitable to calculate the surface of the Young’s and shear moduli of the N-CNTs, for which there is no appropriate value of the NT wall thickness reported in the literature.

The results obtained contribute considerably to a benchmark in the evaluation of the elastic constants of the silicon carbide nanotubes by theoretical methods.

## Figures and Tables

**Table 1:** Input parameters for FE simulations of SWSiCNTs: geometrical and mechanical properties of the beam elements.

Parameter	Value	Formulation
^1^ bond stretching force constant, k_r_ [21]	417 nN/nm	–
^1^ bond bending force constant, k_θ_ [21]	0.842 nN⋅nm/rad^2^	–
^1^ torsional resistance force constant, k_τ_ [21]	1.505 nN⋅nm/rad^2^	–
Si–C bond/beam lengths [22]	0.177 nm	*l* = $a_{\mathrm{Si}\text{-}C}$
diameter, d	0.1797 nm	$d=4\sqrt{k_{\theta }/k_{r}}$
Young’s modulus, $E_{b}$	2937 GPa	$E_{b}{=k}_{r}^{2}l/{4\pi k}_{\theta }$
shear modulus, $G_{b}$	2625 GPa	$G_{b}{=k}_{r}^{2}k_{\tau }l/{8\pi k}_{\theta }^{2}$
Poisson’s ratio, $\nu_{b\;}$	0.29	$\nu_{b\;}=\left(k_{r}l^{2}{-6k}_{\theta }\right)/{(k}_{r}l^{2}{+18k}_{\theta })$
tensile rigidity, $E_{b}A_{b}$	74.5 nN	$E_{b}A_{b}{=lk}_{r}$
bending rigidity, $E_{b}I_{b}$	0.1504 nN⋅nm^2^	$E_{b}I_{b}{=lk}_{\theta }$
torsional rigidity, $G_{b}J_{b}$	0.2688 nN⋅nm^2^	$G_{b}J_{b}{=lk}_{\tau }$

^1^ The force field constants were obtained from DFT’s own calculations combined with molecular mechanics expressions.

**Table 2:** Geometrical characteristics of the studied non-chiral SWSiCNTs.

NT Type	(n, m)	θ°	$\mathrm{Diameter},\;D_{n},\;\mathrm{nm}$
zigzag	(7, 0)	0	0.683
	(11, 0)		1.073
	(14, 0)		1.366
	(17, 0)		1.659
	(21, 0)		2.049
	(26, 0)		2.537
	(31, 0)		3.025
	(36, 0)		3.513
	(40, 0)		3.903
	(43, 0)		4.196
armchair	(4, 4)	30	0.676
	(6, 6)		1.014
	(8, 8)		1.352
	(10, 10)		1.690
	(12, 12)		2.028
	(15, 15)		2.535
	(18, 18)		3.042
	(21, 21)		3.549
	(23, 23)		3.888
	(25, 25)		4.226

**Table 3:** Comparison of the surface Young’s modulus results obtained in the present study with those available in the literature.

Reference	Method	NT Type	$E_{S},\;\mathrm{TPa}\cdot \mathrm{nm}{\;}^{1}$
Baumeier et al. [12]	ab initio	(n, n)	0.167
		(n, 0)	0.162
Setoodeh et al. [14]	MD: Tersoff potential	(n, n)	0.182
		(n, 0)	0.180
Le [17]	MD: harmonic force fields	(n, n)	0.148
		(n, 0)	0.145
Genoese et al. [19]	NCM/MSM + CM: “stick-and-spring” + thin shell models	(n, n)	0.152
		(n, 0)	0.149
Jiang and Guo [20]	NCM/MSM: “stick-and-spring” model + analytical	(n, n)	0.169
		(n, 0)	0.168
Present study	NCM/MSM: beams	(n, n)	0.227
		(n, 0)	

^1^ Converged average value of $E_{S}$ is considered.

## Data Availability

The data presented in this study are available on request from the corresponding author after obtaining the permission of the authorized person.
